# Structural and spectroscopic resolution of the NADPH redox state in the STEAP2 cytosolic oxidoreductase domain

**DOI:** 10.1016/j.jbc.2025.110822

**Published:** 2025-10-14

**Authors:** Inchul Shin, Lu-Zhe Sun, Aimin Liu

**Affiliations:** 1 Department of Chemistry, The University of Texas at San Antonio, San Antonio, Texas, USA; 2 Department of Cell Systems and Anatomy, The University of Texas Health Science Center, San Antonio, Texas, USA

**Keywords:** metalloreductase, X-ray crystallography, NADPH, redox state, single-crystal spectroscopy, electron transfer, prostate cancer, membrane protein, domain reorientation, oxidoreductase domain (OxRD), molecular biophysics

## Abstract

The six-transmembrane epithelial antigen of prostate (STEAP) family of membrane proteins comprises four human metalloreductases essential for iron and copper homeostasis, redox balance, and cell proliferation. These enzymes transfer electrons from cytosolic NADPH to extracellular ferric and cupric ions *via* a FAD and heme-dependent pathway. STEAP2, 3, and four contain an N-terminal cytosolic oxidoreductase domain (OxRD) that enables electron input from NADPH, making STEAP2 ideal for studying redox-state cofactor or cosubstrate dynamics. While recent structures of STEAP proteins have been crucial, the redox state of bound NADPH has remained ambiguous in structural data, limiting mechanistic understanding. Here, we address this key missing piece of ambiguity for understanding the electron transfer pathway. We report high-resolution crystal structures of the STEAP2 OxRD with NADPH, in which the redox state of the cosubstrate is directly validated by single-crystal spectroscopy. Comparison with a recent cryoEM structure reveals conformational differences in the FAD-binding region, suggesting a plausible model in which domain reorientation between the N-terminal OxRD and C-terminal transmembrane domain (TMD) facilitates FADH_2_ loading and FAD release. These findings resolve a key ambiguity in STEAP structural biology and underscore the importance of experimental redox-state verification in structural studies of redox enzymes.

Six-transmembrane epithelial antigen of prostate (STEAP) family comprises a group of highly conserved metalloreductases that play critical roles in various cellular processes, including iron and copper homeostasis, oxidative stress regulation, and cell proliferation (1). Initially identified as an antigen overexpressed in prostate cancer, the STEAP family has been recognized for its broader implications in diverse physiological and pathological conditions (1, 2, 3).

The dysregulation of STEAP proteins has been implicated in a range of diseases. Notably, their association with prostate cancer progression and metastasis has driven significant research interest, positioning them as potential diagnostic biomarkers and therapeutic targets. Beyond prostate cancer, emerging evidence highlights their involvement in other malignancies, metabolic disorders such as obesity and insulin resistance, and inflammatory conditions (3, 4).

There are four known members in the human STEAP family (5). Despite sharing a common structural motif of six transmembrane domains, each member exhibits distinct expression patterns, subcellular localizations, and specific enzymatic activities, leading to specialized biological functions. STEAP proteins are localized primarily to endosomal and lysosomal compartments, where they facilitate the reduction of ferric iron (Fe^3+^) to ferrous iron (Fe^2+^) and cupric copper (Cu^2+^) to cuprous copper (Cu^1+^) (Fig. 1*A*). This reductase activity is crucial for the uptake and utilization of these essential metal ions, which are vital cofactors for numerous enzymes and metabolic pathways.

**Figure 1:**
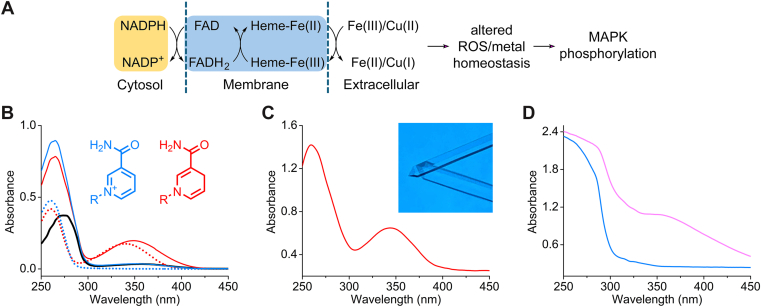
**Spectroscopic determination of the redox state of ligand-bound STEAP2 N-domain.***A*, STEAP functions as metalloreductase. *B*, UV-vis spectra of STEAP2 N-domain (*black*), NADP^+^ (*blue* dotted), NADPH (*red* dotted), STEAP2 + NADP^+^ (*blue* solid), and STEAP2^+^ NADPH (*redsolid*) in solution state. *C*, Single-crystal UV-vis spectrum of STEAP2 in complex with NADPH. The inset shows STEAP2 N-domain crystals. *D*, Single-crystal UV-vis spectra of STEAP2 from a single NADP^+^-soaked crystal. *Blue* trace shows the spectrum taken before any X-ray exposure, and the *pink* spectrum obtained from the same crystal immediately after X-ray diffraction data collection.

STEAP proteins utilize a sophisticated electron transfer chain involving NADPH, FAD, and heme, which ultimately facilitates the reduction of iron or copper. A key structural distinction exists among the family members: STEAP2, STEAP3, and STEAP4 possess a cytosolic N-terminal domain (6). This N-domain serves as the critical starting point for electron transfer within the STEAP metalloreductase pathway, functioning as the primary NADPH binding site, known as the cytosolic oxidoreductase domain (OxRD). In contrast, STEAP1 notably lacks this specific NADPH binding domain, highlighting a divergence in its electron input mechanism. Recent advancements in structural biology, including crystal structures of STEAP3 (7) and rat STEAP4 (8), and cryo-electron microscopy (cryoEM) structures of STEAP1 (9), 2 (10), and 4 (11), have provided valuable insights into their overall architecture. Notably, cryoEM structures of STEAP2 and STEAP4 reveal their trimeric oligomeric state, where the cytosolic NADPH-binding OxRD of one subunit is strategically positioned below the transmembrane domain (TMD) of a neighboring subunit, forming a functional unit for electron transport (10, 11).

Despite these important structural advances, a key ambiguity persists concerning the precise redox state of the bound nicotinamide cofactor. While these structures have provided structural insights, the definitive assignment of the cofactor's redox state remains a significant challenge. The interpretation of electron density for the nicotinamide ring can be ambiguous and is further complicated by the known potential for X-ray or electron beam-induced reduction during data collection. This inherent uncertainty has limited a full mechanistic understanding of the catalytic cycle and underscores the need for direct experimental validation. In this study, we present high-resolution crystal structures of the STEAP2 OxRD bound to NADPH, with the cosubstrate redox state unequivocally confirmed by single-crystal spectroscopy. This combined structural and spectroscopic validation provides the first authentic snapshot of OxRD in its catalytically active form and establishes a foundation for the mechanistic understanding of STEAP-mediated electron transfer.

## Results

### Spectroscopic determination of the redox state of ligand bound STEAP2 OxRD

To determine the redox state of ligand-bound STEAP2 N-domain in the solution state, we performed UV-vis spectroscopy (Fig. 1*B*). STEAP2 N-domain itself shows almost no absorption between 300 and 450 nm (black trace). Free NADPH shows absorption between 300 and 400 nm with a peak centered at 340 nm (red dotted), while free NADP^+^ does not show absorption in this region (blue dotted). Mixing with NADPH and STEAP2 shows absorption with a peak shifted to 350 nm (red). In contrast, STEAP2 with NADP^+^ shows silence in this region (blue). With this firm foundation, we further investigated the redox state of the ligand-bound STEAP2 in the crystalline state. NADPH-bound STEAP2 crystal shows near identical spectroscopic features with its solution state featuring an absorption band centered at 350 nm (Fig. 1*C*). This confirms that the bound ligand in the STEAP2 crystal is NADPH. In contrast, STEAP2 with NADP^+^ in solution shows silence in this region. Similarly, the NADP^+^ soaked crystal before X-ray exposure shows no significant absorption in this region (Fig. 1*D*, blue trace). After X-ray diffraction data were collected, the single-crystal UV-vis spectrum of the NADP^+^ soaked STEAP2 crystal was obtained. Although not identical to Figure 1*C*, the NADP^+^ soaked STEAP2 crystal showed an absorption with a peak at 350 nm (Fig. 1*D*, pink), indicating that the STEAP2 crystal started with NADP^+^, but the reduction occurred during the X-ray diffraction data collection. We further obtained the second X-ray data collection using the identical NADP^+^ soaked crystal.

### Overall structure of NADPH-bound STEAP2 OxRD

**Figure 2:**
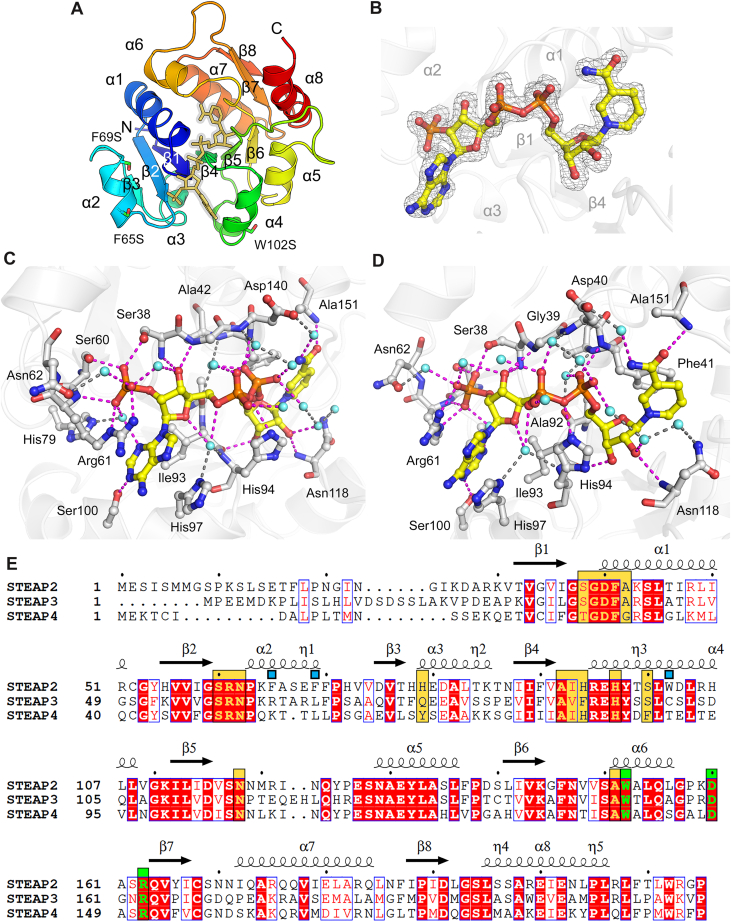
**Spectroscopically verified NADPH-bound crystal structure oftheSTEAP2 cytosolic oxidoreductase domain.***A*, Overall structure. Asymmetric unit contains one protomer of STEAP2 OxRD presented in rainbow color scale from *blue* to *red*, denoting N- to C-termini. NADPH is presented as a *yellow* stick model. The labels of *3*_10_-helices were omitted for clarity. *B*, The Omit *F*_o_–*F*_c_ electron density map of NADPH, contoured at 3 σ. *C* and *D*, H-bond network and the interactions between NADPH and STEAP2 OxRD. Two different views of bound NADPH in STEAP2. *C*, Near adenine nucleotide and 2ʹ-phosphate. *D*, Near pyrophosphate linkage and nicotinamide nucleotide. NADPH is presented in *yellow* carbon color. Water molecules are shown in *cyan* color. Dashed lines with *magenta* color represent the direct interaction of NADPH within 3.2 Å. Indirect interactions of NADPH are presented with *gray* dashed lines. *E*, Sequence alignments of STEAP OxRDs. *Yellow* boxes indicate the residues interacting with NADPH. *Green* boxes indicate the residues' interaction with FAD. *Blue* boxes represent F65S/F69S/W102S triple mutation of STEAP2.

The crystal of STEAP2 OxRD belongs to a primitive hexagonal space group of *P*3_1_21 and contains one protomer in an asymmetric unit (Table 1). The structure consists of 8 β-sheets and 8 α-helices representing the typical Rossmann fold for NADPH binding (Fig. 2*A*). In the cleft running through the middle of the protein, we found an extra unmodelled electron density. With our single-crystal UV-vis spectroscopic data, we confidently assigned NADPH into the density (Fig. 2*B*).

**Table 1:** X-ray diffraction data collection and refinement statistics

Data collection	NADPH soaking	NADP^+^ soaking	NADP^+^ soaking
		First collection^a^	Second collection^b^
Space group	*P3*_1_21	*P3*_1_21	*P3*_1_21
Cell dimension			
a, b, c (Å)	68.2, 68.2, 95.4	68.1, 68.1, 95.2	68.1, 68.1, 95.2
α, β, γ (°)	90, 90, 120	90, 90, 120	90, 90, 120
Resolution (Å)	50.00–1.55 (1.58–1.55)^c^	50.00–1.46 (1.49–1.46)	50.00–1.46 (1.49–1.46)
Total reflections	364,703	443,112	441,018
Unique reflections	37,869	44,810	44,839
*R*_merge_ (%)	9.3 (180.8)	6.0 (118.5)	7.7 (138.3)
I/σI	26.9 (1.1)	43.2 (1.6)	34.3 (1.0)
Completeness (%)	100 (100)	99.5 (97.6)	99.5 (97.8)
Redundancy	9.6 (10.0)	9.9 (9.6)	9.8 (8.9)
CC_1/2_	0.995 (0.471)	0.998 (0.721)	0.998 (0.580)
Refinement			
Resolution (Å)	29.54–1.55	29.49–1.46	29.50–1.46
No. of reflections	37,798	44,754	44,797
*R*_work_/*R*_free_ (%)	19.29/19.96	15.87/18.32	18.73/20.46
No. atoms/*B*-factor (Å^2^)			
Protein^d^	1424/22.9	1424/25.5	1424/24.9
Cofactor	48/20.6	48/23.7	48/23.5
Solvent	234/38.0	240/40.8	236/37.6
RMSD			
Bond length (Å)	0.007	0.007	0.008
Bond angle (°)	1.170	1.166	1.257
Ramachandran statistics^e^			
Favored (%)	97.18	97.74	97.18
Allowed (%)	2.82	2.26	2.82
PDB code	9PGY	9PGX	9PGW

a UV-vis spectra of NADP^+^ soaked crystal were recorded before (Fig. 1*D*, blue) and after first X-ray diffraction data collection (Fig. 1*D*, pink).

b Using the identical NADP ^+^ soaked crystal used for first data collection, second X-ray diffraction data were collected.

c Values in parentheses are for the highest resolution shell.

d Ordered residues: Lys30 – Leu208. The sidechain of Lys64 was omitted due to the lack of electron densities.

e Ramachandran statistics, calculated by MolProbity (17), revealed no outliers.

### STEAP2 and NADPH interactions

NADPH forms an extensive H-bond network with STEAP2 (Fig. 2, *C* and *D*). The 2ʹ-phosphate near the adenine nucleotide forms direct interaction with Ser38, Ser60, and Arg61 within 3.2 Å. The guanidine group of Arg61 π-stacks with the adenine ring of NADPH. Both the ribose ring and pyrophosphate linkage forms largely indirect interactions with STEAP2 *via* water molecules or directly interacts with the mainchain atoms. The nicotinamide ring stacks with Phe41. The carbamoyl nitrogen connects to Asp140 *via* a water molecule, and the carbamoyl oxygen forms a hydrogen bond with the mainchain amide nitrogen of Ala151. Generally, the residues participating in the interaction with NADPH are strictly conserved, except His79, His94, and Ser100 (Fig. 2*E*).

### Comparison with the cryoEM structure of STEAP2

**Figure 3:**
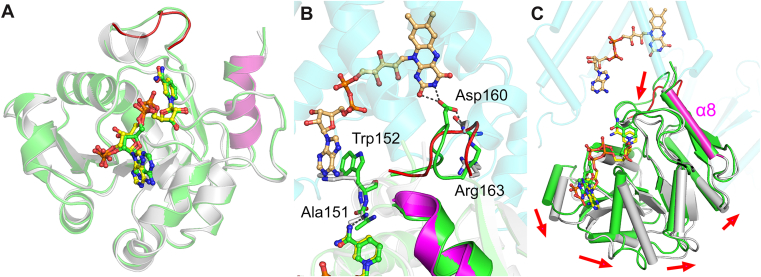
**Comparison with the cryoEM structure of STEAP2 highlights significant conformational differences in the adenine nucleotide moiety of NADP^+^/NADPH and near the isoalloxazine moiety of FAD/FADH_2_, suggesting a conformational transition.***A*, NADP^+^ from the cryoEM structure (*green* and *cyan*) of STEAP2 (7TAI.pdb) is presented with *green* carbon color. NADPH from the crystal structure of STEAP2 OxRD (*gray*) is shown with its carbons in *yellow*. The overall OxRD structures were used for superposition. *B*, FAD is shown in *orange* carbon color. The loop between α6 and β7 in the crystal structure is shown in red. *C*, Superposition using a stable C-terminal end helix α8, ranging 197 to 206 (*magenta*) proposes a plausible domain reorientation anchoring the OxRD to TMD.

The comparison of our crystal structure with the cryoEM structure revealed substantial differences, while the overall structure superposes well with a root-mean-square deviation (RMSD) of 0.63 Å for 177 Cα atoms. The adenine nucleotide of NADP^+^ from the cryoEM structure is flipped and adopts a different conformation (Fig. 3*A*). Other than our crystal structures, the adenine nucleotide moiety adopts a similar flipped conformation from the published STEAP protein structures (Table 2). In the structure comparison with the cryoEM structure of STEAP2, we recognized that there is a significant difference near the isoalloxazine moiety of FAD/FADH_2_ (Fig. 3*B*). The loop between α6 and β7 adopts a significantly different conformation. In the cryoEM structure, Asp160 interacts with the isoalloxazine ring with support by Arg163. In our crystal structure, Asp160 becomes distant to FAD/FADH_2_ and is not stabilized by Arg163. Furthermore, Trp152 is flipped perpendicular in the crystal structure, while Trp152 π-stacks with the adenine nucleotide ring of FAD/FADH_2_ in the cryoEM structure. Investigation of the crystal lattice shows that Trp152 interacts with Pro71. However, the loop region harboring Asp160 and Arg163 faces toward an open space of the crystal lattice without having contact with symmetry-generated neighbors (Fig. S1). The cryoEM structure in this region shows well-resolved densities (Fig. S2), suggesting the difference observed is a biologically relevant intrinsic change. The C-terminal end of OxRD serves as a linker between the N-domain and TMD. Superposition of the crystal structure on the cryoEM structure using the C-terminal end helix α8 (ranging 197–206) presents a noticeable global N-domain movement, suggesting a plausible conformational transition or domain reorientation of OxRD with respect to TMD necessary for the loading of FADH_2_ and unloading of FAD (Fig. 3*C*).

**Table 2:** Summary of the structures of STEAP OxRD

Enzyme	Protein	PDB entryentry	Resolution (Å)	Protein	Method	Reference
STEAP2	OxRD	9PGY	1.55	NADPH	X-ray	This study
	OxRD	9PGX	1.46	NADP^+^ soaking^a^	X-ray	This study
	OxRD	9PGW	1.46	NADP^+^ soaking^b^	X-ray	This study
	OxRD-TMD	7TAI	3.20	NADP^+^	CryoEM	(10)
STEAP3	OxRD	2VQ3	2.00	NADPH^c^	X-ray	(7)
STEAP4	OxRD-TMD	6HD1	3.80	NADPH^c^	CryoEM	(11)
	OxRD-TMD	6HCY	3.10	NADP^+^ (NAP)	CryoEM	(11)

a First collection of NADP^+^ soaked crystal, indicating that a partially reduced NADP^+^ was modeled as NADPH.

b Second collection of the identical NADP^+^ soaked crystal.

c Actual model is NADP^+^ as its 3-letter-code, NAP is used in the PDB entry for this reported structure.

## Discussion

The STEAP family of metalloreductases plays a pivotal role in maintaining cellular metal homeostasis, regulating oxidative stress, and influencing cell proliferation, with dysregulation implicated in various pathologies, particularly prostate cancer. Our current understanding of STEAP protein function relies heavily on its precise enzymatic activity involving NADPH, FAD, and heme within a complex electron transfer chain. NADPH is a proven indispensable component for metalloreductase activity (6). While recent advancements in structural biology, including cryoEM structures of full-length STEAP proteins and crystal structures of their N-domains, have provided valuable insights into their overall architecture and trimeric organization, a critical gap has persisted: the accurate assignment of the cosubstrate’s redox state within these structures based on direct experimental evidence. This ambiguity has hindered a complete mechanistic understanding of electron transfer initiation and the full catalytic cycle.

In this study, we addressed this critical gap by successfully determining the high-resolution crystal structure of the STEAP2 OxRD in complex with its bound cosubstrate. Crucially, our spectroscopic analysis unequivocally established that the bound cosubstrate in both solution and crystalline states of the STEAP2 OxRD is NADPH. This direct experimental evidence provides a firm foundation for our structural determination, allowing us to confidently assign NADPH into the observed electron density within the N-domain's Rossmann fold. Our NADP^+^ soaked crystal clearly exhibited no UV-vis absorption centered at 350 nm, indicating that the bound cosubstrate was indeed NADP^+^ before X-ray exposure (Fig. 1*D*, blue). After collecting X-ray diffraction data, the single-crystal UV-vis spectrum changed, showing absorption at 350 nm (Fig. 1*D*, pink). However, this spectrum was not identical to that of NADPH-bound STEAP2.

One of the key findings is the X-ray-induced reduction of NADP^+^ in the crystal, which is substantial but incomplete, sufficient to influence interpretation. The crystal structures from both the first and second data collections of the NADP^+^ soaked crystal were nearly identical to the NADPH-bound crystal structure, suggesting a partial or near reduction of NADP^+^ to NADPH. While our crystals were prepared with NADP^+^ and showed no signature of NADPH before data collection, they clearly showed a transition to the reduced state after X-ray exposure. This strongly suggests that previous structural studies of STEAP proteins reporting NADP^+^ may have inadvertently characterized a partially or fully reduced cosubstrate and highlights the necessity of direct spectroscopic validation. Any noticeable differences between the crystal structures are probably limited by confined crystalline packing. This finding is significant, as it clarifies the actual redox state, challenging previous structural claims that suggested NADP^+^ binding, which may in fact represent a partially reduced state resulting from X-ray or electron beam-induced reduction during data collection.

Furthermore, our high-resolution structure of the NADPH-bound STEAP2 N-domain reveals a detailed hydrogen bond network and specific interactions crucial for NADPH binding. The strict conservation of most of the interacting residues across the STEAP family underscores the functional importance of these interactions for NADPH binding and electron transfer.

A key finding of our study emerges from the comparison of our NADPH-bound STEAP2 OxRD crystal structure with the previously published cryoEM structures of full-length STEAP2. This comparison revealed substantial differences, particularly concerning the conformation of the adenine nucleotide moiety of NADP^+^ in the cryoEM structure, which appears flipped compared to our NADPH-bound state. More significantly, we identified a marked difference near the isoalloxazine moiety of FAD/FADH_2_, located within a loop between α6 and β7. In the cryoEM structure, Asp160 interacts with the isoalloxazine ring, supported by Arg163. However, in our crystal structure, Asp160 is distant from FAD/FADH_2_, lacking stabilization by Arg163. In addition, Trp152 adopts a perpendicular orientation in our crystal structure, contrasting with its π-stacking with the FAD/FADH_2_ adenine nucleotide ring observed in the cryoEM structure.

The observed conformational changes, particularly the differing orientations of residues near the FAD/FADH_2_ binding site, suggest a dynamic interplay critical for STEAP's catalytic cycle. The C-terminal helix α8 connects to the TMD by a loop and itself shows only minimal differences between the two structures (RMSD of 0.35 Å for 10 Cα atoms). Superimposing our N-domain crystal structure onto the cryoEM structure using helix α8 as a reference revealed a global reorientation of the N-domain, suggesting a hinge-like motion between the N-domain and TMD. We propose that this rearrangement enables efficient loading of FADH_2_ and the subsequent unloading of FAD during the enzymatic cycle. Such a mechanism would facilitate the transfer of electrons from NADPH in OxRD, through the FAD/heme centers, to the metal ions. Notably, it has been established that STEAP2 influences ROS levels and metal homeostasis, leading to MAP kinase signaling in hepatocellular carcinoma (Fig. 1*A*) (12). These structural and biological findings together underscore the significance of a mechanistic framework for how STEAP proteins coordinate electron transfer to drive their catalytic activity.

The findings described in this study provide the first definitive spectroscopic and structural evidence for NADPH binding to the STEAP2 N-domain but also offer critical insights into the conformational dynamics that govern its enzymatic function. These detailed molecular insights are crucial for understanding STEAP-mediated metal homeostasis and its roles in disease.

## Experimental procedures

The detailed process of cloning, expression, and protein purification is included in the Supporting Information. The purified protein was concentrated to 12.3 mg/ml. Crystallization was performed at 22 °C using the sitting drop vapor diffusion method by mixing 1 μl of protein solution with 1 μl of crystallization mother liquor containing 0.1 M Bis-Tris pH 5.5, 2.0 M ammonium sulfate. Crystals were soaked with 10 mM NADPH or NADP^+^ for 1 h at room temperature and flash frozen with liquid nitrogen under the protection of mother liquor containing 30% v/v ethylene glycol. X-ray diffraction data were collected at 100 K at Stanford Synchrotron Radiation Lightsource (SSRL) beamline 9-2. Before exposing the crystal to X-rays, single-crystal UV-vis spectra were obtained using the installed microspec at beamline 9-2. The collected X-ray diffraction data were processed using HKL3000 (13). The phase of the crystal structure was obtained by molecular replacement using the N-domain portion of the cryoEM structure of STEAP2 as a search model using phenix.phaser (14). Iterative model building and refinement were carried out using phenix.refine (14) and coot (15). Structural superpositions were performed using CCP4MG (16). Structure figures were prepared using PyMol (Schrödinger).

Three distinct X-ray diffraction datasets were collected (Table 1). The first dataset was from a crystal soaked with 10 mM NADPH. The second and third datasets were collected sequentially from a single crystal soaked with 10 mM NADP^+^. For this NADP^+^-soaked crystal, a UV-vis spectrum was first recorded before X-ray exposure (corresponding to Fig. 1*D* blue trace). Immediately following this, the first diffraction dataset was collected. A second UV-vis spectrum was then taken from the same spot on the crystal, which showed partial reduction due to the X-ray beam (corresponding to Fig. 1*D* pink trace). Finally, the second diffraction dataset was collected from that same crystal volume.

## Data availability

The crystal structure has been deposited in the RCSB Protein Data Bank with the PDB entries 9PGW, 9PGX, and 9PGY. All other data is contained in the main text and Supporting Information.

## Supporting information

This article contains Supporting Information (Figs. S1 and S2).

## Conflict of interest

The authors declare no financial interests/personal relationships which may be considered as potential competing interests.
