# Supplementary material for: Structural and spectroscopic resolution of the NADPH redox state in the STEAP2 cytosolic oxidoreductase domain
Source: J Biol Chem. 2025 Oct 14;301(12):110822. doi: 10.1016/j.jbc.2025.110822 (PMC12651567; doi:10.1016/j.jbc.2025.110822)
Supplement: Supporting Figures [file mmc1.pdf]

Supporting Information

Structural and Spectroscopic Resolution of the NADPH Redox State in the STEAP2 Cytosolic Oxidoreductase Domain

Inchul Shin[‡], Lu-Zhe Sun[§*], Aimin Liu[‡*]

From the [‡]Department of Chemistry, The University of Texas at San Antonio, Texas 78249, United States and the [§]Department of Cell Systems and Anatomy, The University of Texas Health Science Center, San Antonio, TX 78229, United States

*To whom correspondence may be addressed: Feradical@utsa.edu and sunl@uthscsa.edu

Running Title: *Redox-verified STEAP2 OxRD structures*

Cloning, expression, and protein purification

STEAP2 N-domain ranging (residues Lys30 - Leu208) was amplified by PCR using primers:

forward, 5′-GCTCATATGAAGGTCACTGTAG-3′
reverse, 5′-GCTAAGCTTCTAGAGTCGTAGGG-3′

The amplified gene was subcloned into pET-28a-TEV vector between NdeI and HindIII restriction enzyme sites. DNA sequence was verified by DNA sequencing (Eurofins Genomics).

The expressed protein presents a solubility issue. We inspected the protein surface and identified three hydrophobic residues exposed to the solvent front: Phe65, Phe69, and Trp102. These residues are not conserved in STEAP3 - 4 and are located distant from the bound cosubstrate, not being involved in cosubstrate binding. Therefore, these residues are not expected to alter the function of cosubstrate binding. We introduced triple mutation, F65S/F69S/W102S using site-directed mutagenesis PCR: F65S/F69S forward primer, 5′-GAAATCCTAAGTCTGCTTCTGAATCTTTTCCTCATGTG-3′; W102S forward primer, 5′-ATTATACCTCCCTGTCGGACCTGAGACATCT-3′; reverse primers are the reverse complement of the forward primers. The *Escherichia coli* BL21(DE3) was transformed with the resultant construct. Cell culture was conducted at 37 °C, 220 rpm using LB medium with 50 μg/mL kanamycin. Gene expression was initiated by adding IPTG when optical density at 600 nm reached 0.8 and the induction was carried out at 20 °C for 20 h. Harvested cells were resuspended with 50 mM Tris-HCl, 200 mM NaCl, 20% v/v glycerol (pH 8.0): 100 mL per 10 g of cell pellet. The cell membrane was disrupted by Microfluidizer LM-20 (Microfluidics). Cell debris were removed by centrifugation at 36,000 *g* for 2 h at 4 °C.

The supernatant of cell lysate was applied to HisTrap column (Cytiva). The target protein was eluted with the buffer containing 50 mM Tris-HCl, 200 mM NaCl, 500 mM imidazole, 20% v/v glycerol (pH 8.0). The N-terminal His-tag was removed by treatment with tobacco etch virus (TEV) protease. The untagged protein was separated from TEV protease by applying it to HisTrap column. The flow-through fraction contained our target proteins and was further purified by size-exclusion chromatography using a Superdex 75 column with a buffer containing 10 mM Tris-HCl, 150 mM NaCl, 10% v/v glycerol, 0.5 mM β-mercaptoethanol (pH 8.0). The concentration of the purified protein was measured by UV-vis spectroscopy. The extinction coefficient at 280 nm, 13,075 $M^{-1}cm^{-1}$ and molecular weight 20,240.27 Da were calculated using Expasy ProtParam (https://web.expasy.org/protparam/) and used for concentration measurement.

Spectroscopy

Agilent 8453 UV-visible spectrophotometer with a diode array detector was used. NADPH and $NADP^+$ were dissolved in buffers containing 10 mM Tris-HCl, 150 mM NaCl, 10% v/v glycerol (pH 8.0).

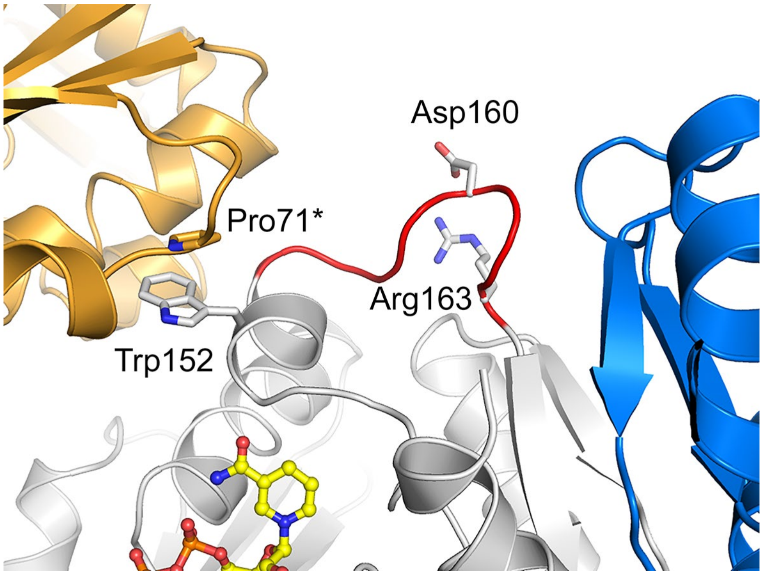

Figure S1. Analysis of the crystal packing environment around the region of conformational difference. The central STEAP2 OxRD protomer is shown in gray cartoon representation. The loop containing residues Asp160 and Arg163, which exhibits the largest conformational difference compared to the cryoEM structure, is highlighted in red. Two adjacent, crystallographic symmetry-generated protomers are shown in gold and blue. The red loop is fully solvent-exposed and does not participate in any crystal packing contacts, indicating its conformation is not a lattice-induced artifact. A minor crystal contact is observed between Trp152 of the central protomer and Pro71* of the adjacent gold subunit, which is distant from the main flexible loop.

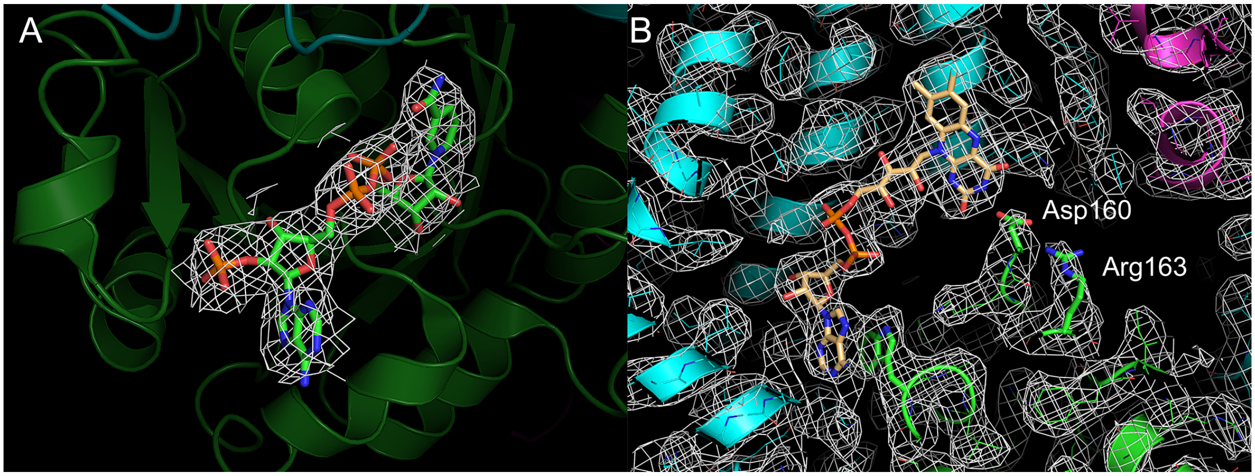

Figure S2. Analysis of the cryoEM density map in regions of conformational difference. *A*, The cryoEM density map (EMD-25775) is shown as a grey mesh contoured at 5.0 σ. *B*, The densities for the FAD cofactor (orange carbons) and the interacting loop residues Asp160 and Arg163 are also well-resolved, confirming their conformation in the cryoEM structure. The OxRD (green), TMD of the adjacent subunit (cyan), and TMD of the third subunit (magenta) are shown for context.
